# The effect of traditional Chinese medicine on gut microbiota in adults with type 2 diabetes: A protocol for systematic review and meta-analysis

**DOI:** 10.1097/MD.0000000000022233

**Published:** 2020-09-18

**Authors:** Yujiao Zheng, Qiyou Ding, Lili Zhang, Xiaowen Gou, Yu Wei, Min Li, Xiaolin Tong

**Affiliations:** a Graduate School, Beijing University of Chinese Medicine; b Department of Endocrinology, Guang’anmen Hospital, China Academy of Chinese Medical Sciences; c Molecular Biology Laboratory, Guang’anmen Hospital, Chinese Academy of Chinese Medical Sciences, Beijing, China.

**Keywords:** gut microbiota, protocol, systematic review, traditional Chinese medicine, type 2 diabetes

## Abstract

**Background:**

Type 2 diabetes (T2DM), which is the major type of diabetes, accounts for more than 90% of all case of diabetes, and its pathogenesis remains inconclusive. Recent studies have revealed a significant role of gut microbiota in the onset and development of T2DM. Traditional Chinese medicine (TCM) has accumulated rich clinical experience in the treatment of T2DM for thousands of years and a large amount of studies have shown that TCM has the capacity of lowering blood glucose and modulating gut microbiota. The aim of this systematic review is to evaluate all randomized controlled trials on TCM for gut microbiota to assess the effectiveness and safety of TCM in T2DM patients.

**Methods:**

Seven electronic databases (Web of Science, PubMed, EMBASE, Cochrane Library, China National Knowledge Infrastructure, Wanfang Database, and VIP Information-Chinese Scientific Journal Database) will be searched from inception to present in the English and Chinese languages. Eligible randomized controlled trials evaluating the effect of TCM in T2DM patients, compared with western medicine, placebo or no intervention will be included in the study. The primary outcomes are the glucose metabolism and gut microbiota as well as its metabolites. The second outcomes are changes in weight, and changes in inflammatory markers. Two authors will independently select studies, extract data, and assess the quality of the studies by scanning the titles, abstracts, and full texts. The meta-analysis will be conducted using Review Manager version 5.3. The results will be presented as risk ratios for dichotomous data and adverse events, and as mean differences for continuous data.

**Result:**

The study will provide a summary of current evidence for the treatment of T2DM from the perspective of gut microbiota by using TCM based on the outcome measures.

**Conclusion:**

The systematic review will evaluate the efficacy of TCM in treating T2DM from the perspective of gut microbiota, providing current evidence and laying a foundation for further work in the field.

**PROSPERO registration number:**

CRD42020188043.

## 1 Introduction

As one of the major chronic diseases, diabetes has brought a huge healthcare burden to healthcare systems worldwide. According to the latest data from the International Diabetes Federation, approximately 463 million (9.3%) people suffered from diabetes in 2019, and the prevalence will rise to 578 million (10.2%) by 2030 and 700 million (10.9%) by 2045.^[1]^ Type 2 diabetes mellitus (T2DM), which is the main type of diabetes, has become a major public concern worldwide, it is estimated to account for more than 90% of all case of diabetes.^[2]^ Most patients with T2DM have at least 1 complication, mainly microvascular and macrovascular complications. Among them, cardiovascular complications are the leading cause of morbidity and mortality in T2DM patients.^[3]^ The pathogenesis of T2DM remains inconclusive. Nationwide studies have shown that genetics and environmental effects play a significant role.^[4]^ Lifestyle factors, particularly factors associated with obesity, also contribute significantly to the onset and development of T2DM.^[5]^

During the past decade, accumulated evidences have supported an increasingly more important role of gut microbiota in obesity, glycemic control, and T2DM.^[6]^ Gut microbiota, which comprise a complex ecological community in the human body, play a vital physiological role in biological processes such as digestion, vitamin synthesis, and metabolism amongst others.^[7]^ Modern pharmacology studies have revealed that the mechanisms of diabetes have a close relationship with gut microbiota, including the aspects of short-chain fatty acid (SCFA) production, endotoxemia, low-grade inflammation, intestinal mucosal permeability changes, interaction with bile acids, brown adipose tissue, and therapeutic effects of some hypoglycemic agents.^[8–10]^

Traditional Chinese medicine (TCM), which has a history of thousands of years in China, is the primary branch of the alternative and complementary medicine and the unique cultural treasure for the Chinese. From ancient times to the present, TCM has accumulated rich clinical experience in the treatment of T2DM. A large amount of studies have shown that TCM has the capacity of lowering blood glucose, improving inflammation and modulating gut microbiota.^[11–13]^ Chinese herbal medicine (CHM), the main component of TCM, is usually rich in fiber and phytochemicals. When an anti-diabetic CHM is taken orally, its bioactive components are absorbed in the intestine and change the composition of gut microbiota, including inhibiting pathogens such as *Escherichia coli*, and promoting beneficial bacteria such as *Adlercreutzia, Alloprevotella*, *Fecalibacterium prausnitzii*, *Lactobacillus,* and *Bifidobacterium*.^[14–17]^ In addition, CHM can improve blood glucose through affecting the metabolites of gut microbiota. For instance, they can increase SCFA production, and an increasing SCFA level is closely related to GLP-1 secretion and energy metabolism.^[18,19]^

Despite an amount of reviews summarizing the effect of TCM in relation to gut microbiota and T2DM have been published in recent years and many related clinical trials have been undertaken,^[20]^ no systematic review of the impact of TCM on T2DM from the perspective of gut microbiota modulation has been conducted. The aim of this study is to systematically review randomized controlled trials (RCTs) to assess the evidence of the efficacy of TCM on gut microbiota in patients suffering from T2DM.

## 2 Methods

### 2.1 Eligibility criteria

The inclusion and exclusion criteria for the review are based on population, intervention, comparison, outcome and study type (PICOS) acronym. We aim to select RCTs (S) evaluating the effect (O) of TCM (I) compared with that of western medicine, placebo or no intervention (C), in adults with T2DM (P). The inclusion and exclusion criteria are listed in Table 1.

**Table 1:**
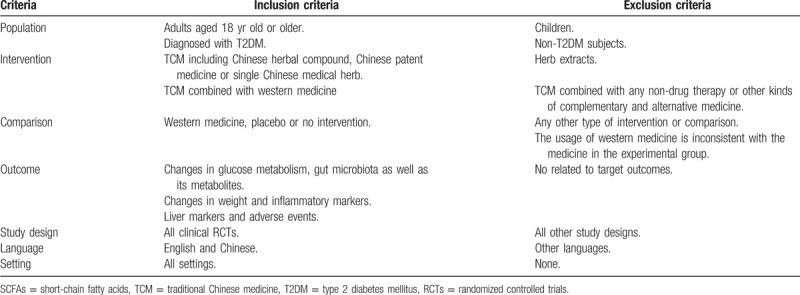
Inclusion and exclusion criteria.

#### 2.1.1 Types of studies

All human RCTs investigating the TCM intervention in the treatment of adults suffering from T2DM will be included in the English and Chinese languages.

#### 2.1.2 Types of participants

Adult participants with T2DM will be included in this review. The diagnosis of T2DM will be based on the 1999 World Health Organization criteria (fasting plasma glucose [FPG] ≥7 mmol/L and/or 2-hour oral glucose tolerance test [2-h OGTT] ≥11.1 mmol/L).

#### 2.1.3 Types of interventions and comparators

In the included studies, the intervention received by adults with T2DM in the experimental group will be TCM including Chinese herbal compound, Chinese patent medicine, and single Chinese medical herb. The TCM intervention can be administered in the form of decoction, granule, or powder. Studies on herb extracts will be excluded. Besides, TCM combined with any non-drug therapy or other kinds of complementary and alternative medicine will also be excluded. The control group will receive either western medicine, placebo, or no intervention. If TCM is combined with western medicine in the experimental group, the use of western medicine will have to be consistent with that of the control group.

#### 2.1.4 Outcomes

We will search for all published quantitative research based on outcome measures of glucose metabolism (glycated hemoglobin, FPG, 2-h OGTT, serum C-peptide, serum insulin) and gut microbiota as well as its metabolites (faecal metabolome and ribosomal RNA sequencing), as they are the primary outcomes. Other outcome measures related to the target conditions will also be considered, including, but not limited to, changes in:

1. weight as measured via waist circumference, body mass index and weight.
2. inflammatory markers (such as tumor necrosis factor alpha, C-reactive protein, IL-6, and IL-1B).

Besides, other outcomes including liver markers (fasting serum aspartate aminotransferase and alanine aminotransferase) and the adverse events during the treatment period will also be included to evaluate the safety of TCM intervention.

### 2.2 Search methods for study identification

#### 2.2.1 Data searches

Seven electronic databases (Web of Science, PubMed, EMBASE, Cochrane Library, China National Knowledge Infrastructure, Wanfang Database and VIP Information-Chinese Scientific Journal Database) will be searched from inception to present in the English and Chinese languages. Reference lists in the identified articles and reviews, as well as studies that cited these articles, will be searched with Scopus. We will also search the gray literature via trial registries and conference papers. If the published articles provide inadequate data, we will attempt to contact the corresponding author for additional information.

#### 2.2.2 Search strategy

The search strategy will be as follows: (“type 2 diabetes” or “type 2 diabetes mellitus” or “T2D” or “T2DM”) and (“gut microbiota” or “gastrointestinal microbiome” or ((“faecal” or “fecal”) and (“bacteri∗” or “flora” or “microbio∗”)) or “gut flora” or “dysbiosis” or “eubiosis”) and (“traditional Chinese medicine” or “TCM” or “Chinese herbal medicine” or (“Chinese” and (“medicine” or “decoction” or “formula” or “prescription” or “drug”)) or “Chinese medicinal herb” or “Chinese patent medicine” or “Chinese herbal compound prescription”) and (“randomized controlled trial” or “controlled clinical trial” or “random∗” or “trial” or “RCT”).

### 2.3 Selection of studies

**Figure 1:**
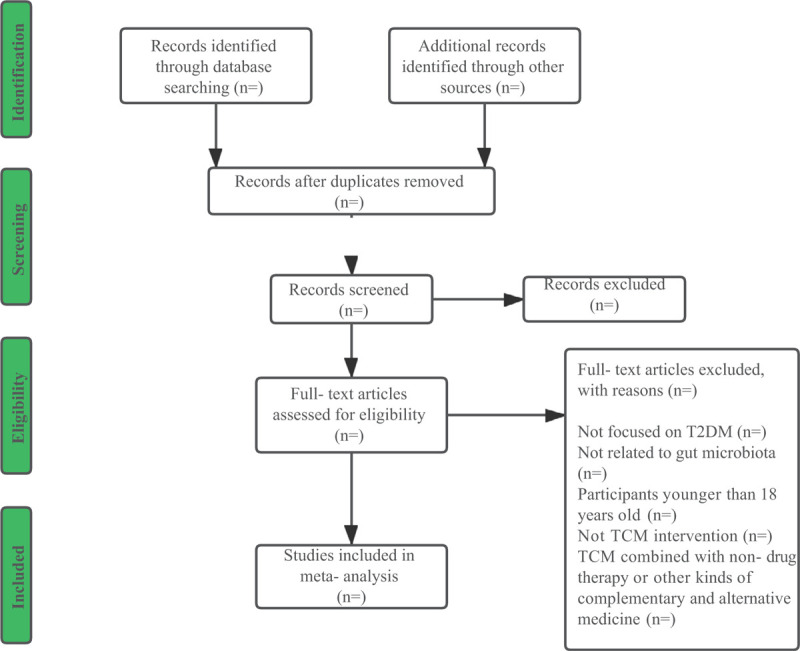
Flow diagram of study selection process.

The study selection process is summarized based on preferred reporting items for systematic reviews and meta-analyses guidelines (PRISMA). Two authors (YZ and QD) will manually and independently select the relevant literature by screening titles and abstracts, followed by assessing the full text based on the eligibility criteria. Duplicates will be removed, and reasons for study exclusion will be recorded. Any disagreements will be resolved through discussion with the third author (LZ). The authors of the trials will be contacted for clarification when necessary. A flowchart of the selection process is shown in Figure 1.

### 2.4 Data extraction and management

Two authors (YZ and QD) will extract and manage the data on population, sample size, participants’ characteristics (age, sex, disease duration, body mass index, etc), study design, randomization, TCM intervention and dosage, duration of intervention, control group, baseline gut microbiota profile/blood glucose control/inflammatory biomarkers, treatment outcomes, adverse events, and other information. Another author (WG) will double-check the extracted data. Resolution of any disagreements will be achieved through discussion with the help of the third author (LZ). When the relevant data are missing or inadequate, the corresponding author of the study will be contacted by e-mail or telephone.

### 2.5 Risk of bias assessment

The risk of bias (ROB) of all included studies will be evaluated independently by 2 authors (YZ and LZ) using the Cochrane Collaboration's tool. If there are any disagreements, we will discuss with a third author (YW). The assessment of ROB will be based on the following aspects: random sequence generation, allocation sequence concealment, blinding of participants, blinding of outcome assessment, incomplete outcome data, selective outcome reporting, and other sources of bias. The results of ROB assessment will be classified as low, high, or unclear risk.

### 2.6 Data synthesis and statistical analysis

The software provided by the Review Manager version 5.3 (The Nordic Cochrane Centre, The Cochrane Collaboration; Copenhagen, Denmark) will be used to analyze the data. If sufficient RCTs (≥ 2) with robust heterogeneous pooled data for each outcome are identified, a meta-analysis will be conducted. Dichotomous data and the adverse events will be reported as risk ratios with 95% confidence intervals. The mean difference will be presented with 95% confidence interval to evaluate the continuous outcomes. Expected inconsistencies across studies will be evaluated using the x^2^ test and I^2^ test. If there is no significant heterogeneity (*P* > .10 and I^2^ < 25%), a fixed-effect model will be used for data analysis. If *P* < .10 and I^2^≥50%, the heterogeneity will be considered to be substantial, its sources will be further analyzed, and a random effect model will be used. Obvious clinical heterogeneity (I^2^≥50%) will be treated by subgroup analysis, sensitivity analysis or narrative analysis. In addition, if insufficient RCTs are available for meta-analysis, we will still complete a narrative synthesis of the included studies, summarizing the study characteristics and TCM effectiveness based on the specific results of the included studies.

### 2.7 Subgroup analysis

If significant heterogeneity is observed in the studies, subgroup analysis will be conducted according to participant characteristics, outcome measures, the composition of TCM and control interventions, depending on the number of selected studies and their sample size.

### 2.8 Sensitivity analysis

A sensitivity analysis will be conducted based on the sample size, the impact of missing data and the methodological quality of the included studies. If necessary, we will exclude low-quality studies and repeat the meta-analysis to test the robustness of the pooled results.

### 2.9 Reporting bias

If there are more than 10 trials included in the study, visual asymmetry on a funnel plot will be utilized to detect reporting bias and the funnel plot asymmetry will be evaluated with the use of Egger test.

### 2.10 Grading evidence quality

The quality of evidence for the outcomes will be evaluated using the grading of recommendations assessment, development and evaluation approach (GRADE). The evaluation will be include the following aspects: ROB, heterogeneity, precision, directness, and publication bias. Every evidence will be classified as high, moderate, low, or very low.

### 2.11 Registration

This study has been registered in the International Prospective Register of Systematic Reviews with the registration number of CRD42020188043. This protocol adheres to the preferred reporting items for systematic reviews and meta-analysis protocols 2015.^[21]^ A standard version of the protocol has been registered with the International Prospective Register of Systematic Reviews https://www.crd.york.ac.uk/prospero/display_record.php?RecordID=188043, and will be updated as necessary.

### 2.12 Ethical approval and dissemination

Since no individualized data will be used in this study, the requirement for formal ethical approval is not necessary. The dissemination of the review findings will include peer-reviewed publications (print and online) and conference presentations.

### 2.13 Patient and public involvement

No patients or public will be involved in this protocol.

## 3 Discussion

As one of the effective therapies for the treatment of T2DM, TCM has the advantages of having multiple targets and fewer side effects. With the in-depth exploration of gut microbiota and the related mechanisms in recent years, TCM has been reported to be effective against diabetes via the regulation of gut microbiota. Many related clinical trials and basic experiments are also being carried out. However, as far as it can be established, there have been no systematic reviews of RCTs on the effects of TCM in the treatment of T2DM from the perspective of gut microbiota. Therefore, we believe it is necessary to conduct a systematic review to provide a summary of current evidence, which will lay a foundation for further work in the field. The findings of the study will contribute to the clinical management of T2DM as well as help to elucidate the therapeutic effect of TCM on T2DM and the mechanisms behind.

## Author contributions

**Conceptualization:** Xiaolin Tong, Min Li.

**Data curation:** Lili Zhang, Xiaowen Gou, Yu Wei.

**Formal analysis:** Yujiao Zheng, Qiyou Ding, Lili Zhang.

**Funding acquisition:** Min Li.

**Investigation:** Xiaowen Gou, Yu Wei.

**Methodology:** Yujiao Zheng, Qiyou Ding, Lili Zhang, Yu Wei.

**Project administration:** Xiaolin Tong, Min Li.

**Software:** Xiaowen Gou.

**Supervision:** Xiaolin Tong, Min Li.

**Validation:** Xiaolin Tong, Min Li.

**Writing – original draft:** Yujiao Zheng, Qiyou Ding.

**Writing – review & editing:** Yujiao Zheng, Qiyou Ding, Lili Zhang.

## Abbreviations

Abbreviations: CHM = Chinese herbal medicine, RCT = randomized controlled trial, ROB = risk of bias, SCFA = short-chain fatty acid, TCM = traditional Chinese medicine, T2DM = type 2 diabetes.
